# Compatibility assessment of a temperature-controlled radiofrequency catheter with a novel electroanatomical mapping system

**DOI:** 10.3389/fcvm.2023.1086791

**Published:** 2023-04-12

**Authors:** Luigi Pannone, Ivan Eltsov, Robbert Ramak, David Cabrita, Paul De Letter, Gian-Battista Chierchia, Carlo de Asmundis

**Affiliations:** ^1^ Heart Rhythm Management Centre, Postgraduate Program in Cardiac Electrophysiology and Pacing, Universitair Ziekenhuis Brussel—Vrije Universiteit Brussel, European Reference Networks Guard-Heart, Brussels, Belgium; ^2^ Medtronic Inc., Minneapolis, MN, United States; ^3^ Abbott Laboratories, St Paul, MN, United States

**Keywords:** catheter ablation, universal compatibility, diamondTemp ablation system, ensite electroanatomic mapping system, cardiac arrhythmias

## Abstract

**Background:**

The novel DiamondTemp ablation system (DTA) and EnSiteX mapping System (EAM) are both CE-Marked and FDA approved medical devices. The DTA has been validated by its manufacturer only in combination with previous version of EnSite System—EnSite Precision. The aim of this study was to evaluate compatibility of DTA with EnSite X with a previously developed protocol.

**Methods:**

Three configurations were tested: 3.1. Medtronic Generator connection Box (GCB) and AmpereConnect cable; 3.2. the Medtronic GCB-E and electrogram out cable from GCB to EAM; 3.3. Direct connection of DTA to EAM using intracardiac out cable with no GCB.

**Results:**

The previously developed universal method for compatibility assessment of ablation catheters and navigation systems was used with success for assessing DTA and EnSite X EAM compatibility, with reproducible results. Accuracy of DTA visualization with different setups was evaluated with a phantom model measuring distances between DTA and reference points. DTA is compatible with EnSiteX EAM with a safety and reliability profile guaranteed, if within the described specifications. In particular, careful setup is mandatory to achieve good clinical outcomes as only setup 3.2 is viable for both NavX and Voxel Mode and demonstrated satisfactory results and accuracy. Setup 3.3 showed a significant shift immediately after catheter insertion. Catheter position was away from baseline points and the dislocation increased during the radiofrequency delivery.

**Conclusions:**

Previously developed method for compatibility assessment of ablation catheters and navigation systems has been used for a new EAM. DTA is compatible with EnSiteX EAM with proper configuration.

## Introduction

Fast development of the medical device industry in the last decades has led to novel technologies from different manufacturers. Electrophysiology products are one of the most dynamic parts of the entire medical device market and the cross-compatibility between electrophysiological devices is not always investigated. As compatibility is not guaranteed “out of the box” the need for compatibility assessment methods between devices from different manufacturers becomes crucial.

Our group has previously developed a novel universal compatibility assessment method to evaluate the safety and accuracy of a new temperature-controlled radiofrequency (RF) catheter ablation system with 3rd party electro anatomical mapping systems (1).

The aim of this research is to the apply our bench testing protocol to assess the compatibility between a temperature-controlled RF catheter ablation and a novel Electroanatomical mapping system (EAM), EnSiteX^TM^ (Abbott, St. Paul, MN).

EnSiteX^TM^, offers both a primary impedance mode (NavX) and a primary magnetic mode (Voxel) (2).

Magnetic based systems are constantly sending localization information based on magnetic field measurements, which allows the system to track it and represent it in the 3D model, but it is possible only for catheters manufactured by the same company.

Impedance-based tracking allows to visualize and tracking virtually any catheter using the impedance measurement between external patches and an electrode on the catheter. However, the precision of this tracking needs to be assessed. Hybrid tracking of EnSiteX system in Voxel mode uses both magnetic and impedance-based methods, where magnetic tracking is enhancing localization accuracy of impedance tracked catheter by acquisition of Voxels.

The novel DiamondTemp^TM^ ablation system (DTA), (Medtronic Inc, Minneapolis, MN) and EnSiteX^TM^ EP System (Abbott, St. Paul, MN) are both CE-Marked and FDA approved medical devices (3, 4). The DTA has been validated by its manufacturer only in combination with previous version of EnSite System—EnSite Precision^TM^ System (Abbott, St. Paul, MN) (5). The aim of this study was to evaluate compatibility between DTA and EnSiteX with a previously developed protocol (1). Furthermore, different configurations have been tested to ensure accuracy of DTA visualization in the novel mapping system.

## Methods

### Ablation catheter and mapping system

**Figure 1:**
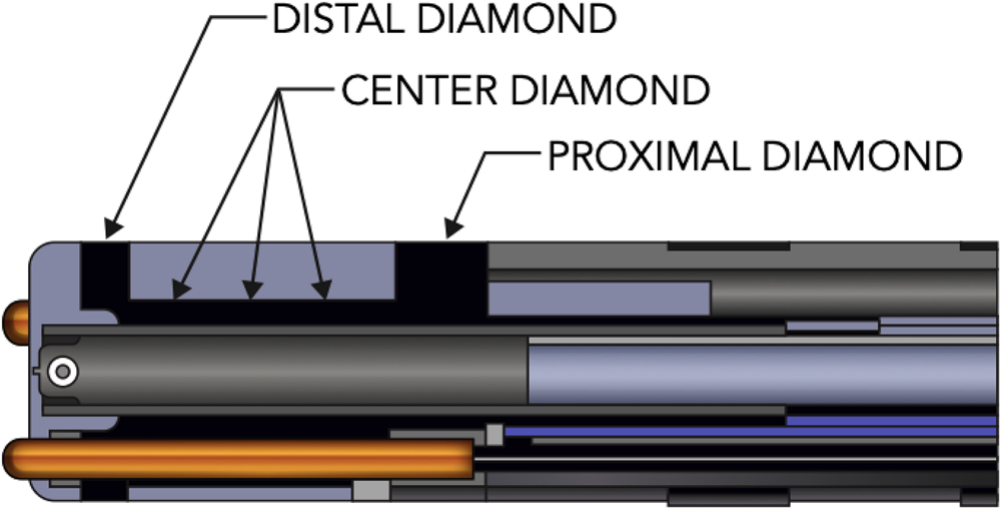
Diamondtemp^™^ catheter composite tip structure. The tip of the catheter consists of a diamond network—distal, proximal and center diamond. Three thermally isolated thermocouples protrude from the distal tip to ensure enhanced tip-tissue interface temperature readings; another 3 thermocouples are embedded in the proximal part to allow correct measurements.

The DTA ablation catheter is a 7.5-F irrigated RF catheter; the 4.1-mm composite tip electrode delivers RF. The ablation electrode tip is embedded with 3 interconnected diamonds, which allows rapid RF delivery (due to electric and thermal diamond properties) and shunting heath from externalized thermocouples, Figure 1. This allows accurate temperature measurement at tip-tissue interface. The catheter operates in temperature control mode and a dedicated RF generator (RFG), titrates rapidly the delivered power to the target temperature. The dual composite ablation tip behaves as a single electrode during ablation and electrically insulation of the tip allows for high-resolution EGM sensing (3, 4).

**Figure 2:**
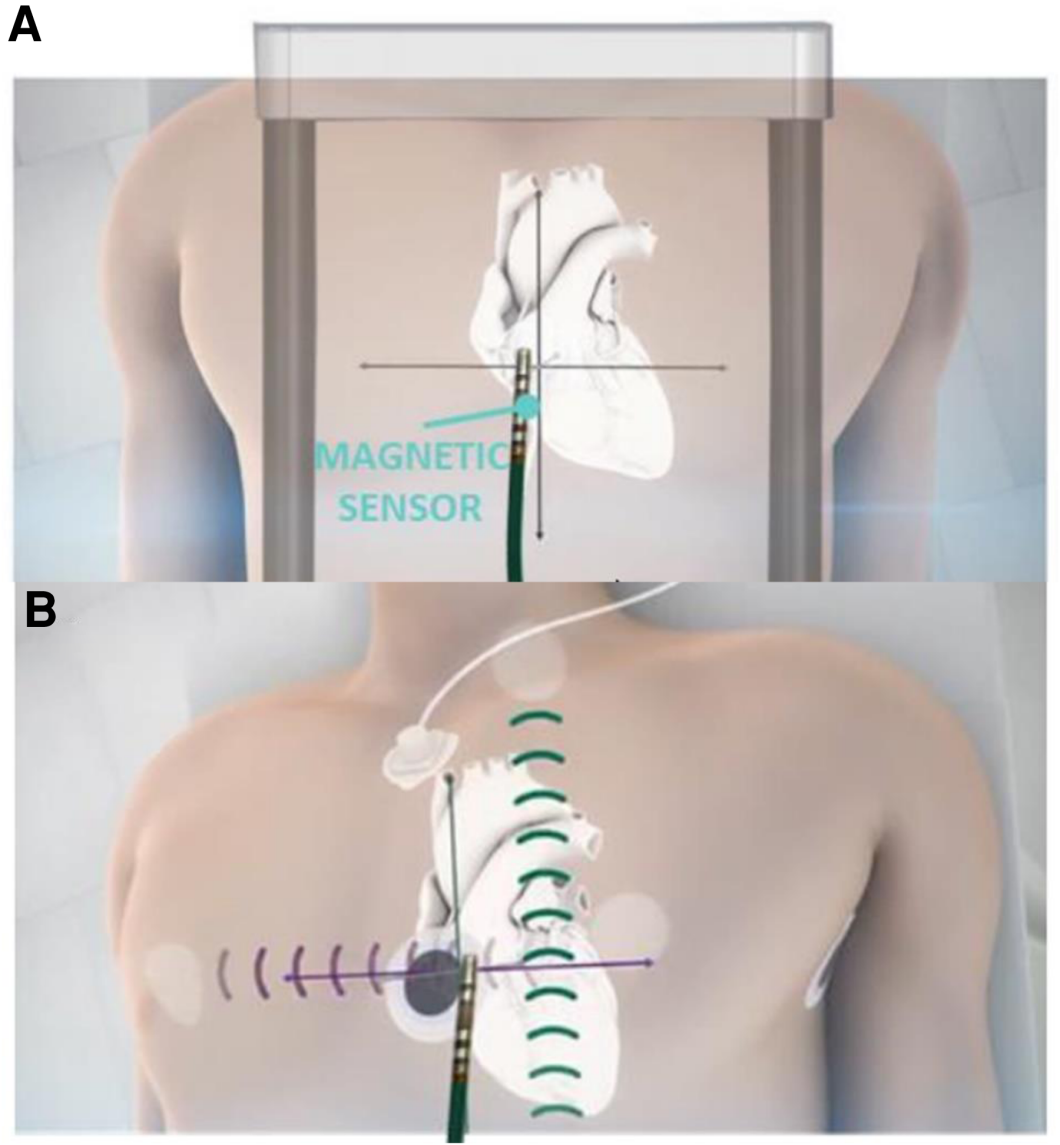
Ensite X voxel and NavX modes tracking. Panel **A**: schematic mechanism of magnetic based tracking of sensor enabled (SE) catheters (Voxel Mode). Panel **B**: impedance-based visualization of catheters without magnetic sensor (NavX mode).

EnSite X^TM^ EP System is a novel EAM which is result of further development of EnSite Product Family. The system can operate in 2 different modes—Voxel mode and NavX mode. NavX mode is an enhanced version of the previous feature of the EnSite™ Precision Cardiac Mapping System. Catheter location is purely based on impedance data, Figure 2. Magnetic data is optional and used to improve impedance tracking. Tracking is also dependent on local changes in thoracic impedance (lungs, sheath, etc.). In Voxel Mode the inflexible portion of sensor enabled (SE) catheter location is based on magnetic localization, Figure 2. The system collects Voxels (Impedance Fiducials) which allow Impedance-based electrodes to be shown. SE catheter location is based on impedance & magnetic data. Catheter shape is consistent despite local impedance changes. Non-SE catheters are visualized using Voxels as well which allows better precision compared to NavX mode, Figure 2.

### Assessment endpoints

This study has 3 endpoints

1) Safety and efficiency of RF energy delivery when a specific setup is used. To ensure that RF energy and current emitted by the system does not leak throughout the setup and can be entirely delivered to the tissue.
2) The DTA is correctly represented inside the 3D model, with a precise and reliable position not influenced by external factors, especially RF energy delivery. Four different configurations have been tested.
3) Reproducibility of results, in comparison with earlier testing according to the described compatibility assessment method (1).

The DTA can be connected to the navigation system either by using electrogram (EGM) out cables from RF generator or from dedicated GenConnect interface to be able to filter out RF energy so it does not affect localization. Previous version of EnSite has been validated to be used in combination with Genconnect connected to the EAM directly—so this configuration was tested as well. In addition, the new EnSite X system has 2 operating modes NavX and Voxel mode. As the DTA can be visualized using impedance mode—testing have been performed in NavX mode. Compatibility of DTA with EnSiteX in VoXel mode has been also assessed for investigational purposes, however qualificative data is not available as in this mode it is impossible perform reliable measurements, so results of this evaluation can be considered only speculative.

**Figure 3:**
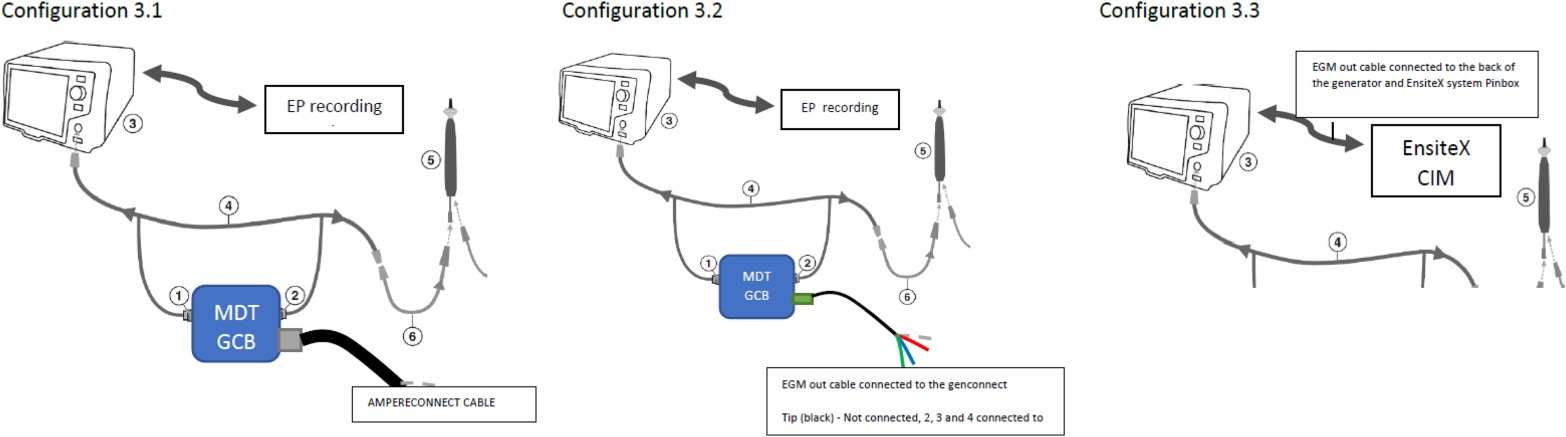
Test configurations (setups). Configuration 3.1—DTA connected to the EnsiteX *via* AmpereConnect cable connected to the MDT Genconnect. Configuration 3.2—DTA connected *via* EGM out cable from the genconnect to the Ensite CIM (without distal as it is not wired). Configuration 3.3 EGM cable from RFG back to CIM and not from genconnect.

**Test configurations (setups),** Figure 3**:**

**Figure 4:**
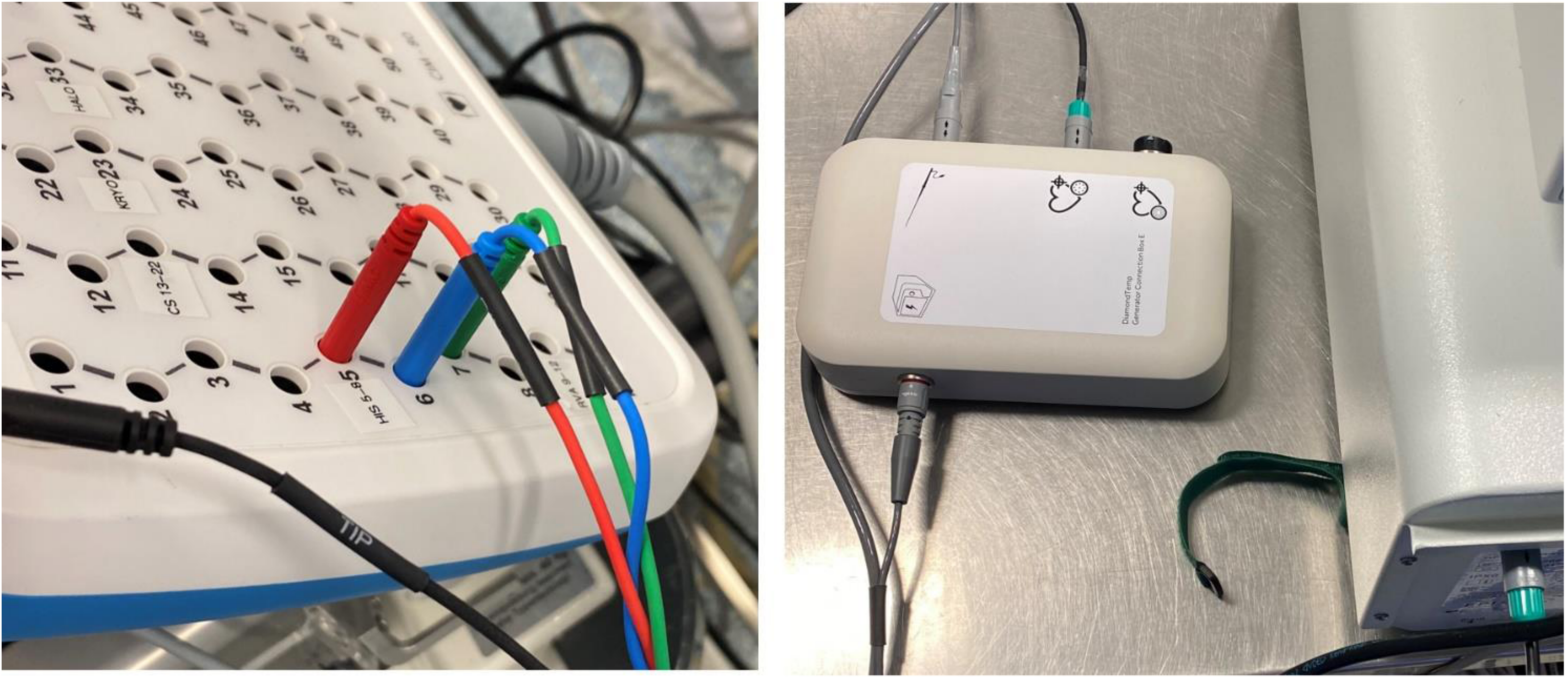
Configuration 3.2 details. Electrogram (EGM) out cable connected to the Genconnect and to the EnsiteX CIM pins 2–4.

1.0. DTA connected only to the DTA RFG—No GenConnect (GC) nor Genconnect Cable (GCC) connected to the DTA;
2.0. DTA not connected to a MS but connected to:
  2.1. The Medtronic (MDT) Generator Connection Box E (MDT GCB-E)
  2.2. The MDT GCB-E and intracardiac (IC) out *via* the MDT GCB-E;
3.0. DTA connected to the EnSiteX EAM using:
  3.1. MDT GCB and AmpereConnect cable
  3.2. The MDT GCB-E and EGM out cable from GCB to EAM (Figures 3, 4)
  3.3. The direct connection of DTA to EAM using IC out cable with no GCB.

Configurations 1.x and 2.x were used for functional and safety assessment only.

### Functional and safety parameters assessment

To assess functional and safety parameters of the DTA with the different setups proposed, a calibrated electrosurgery analyzer “FLUKE Biomedical QA-ESII” was used (6). This device allows for continuous measurement of power, current, peak-to-peak voltage (closed load only) and crest factor for each RF application. The test and connectivity of the FLUKE Biomedical QA-ESII Electrosurgery Analyzer equipment to different components is performed in “continuous operation mode” with no footswitch. The test is interrupted by pressing the “stop” key. The Analyzer acts like a meter during the test, showing increasing and decreasing values as received from the unit being tested, in this case the DTA RFG. The DTA RFG is connected to the connection for the electrode outputs of an internal variable resistance. An active connection (Red) is directly connected to the catheter tip alligator clip wired to Red pin. The neutral connection is direct wired from the DTA neutral plate connection and the analyzer neutral pin (Black).

The functional and safety parameters of the DTA and its RFG were assessed at 3 different loads, namely 50 Ohms, 100 Ohms and 150 Ohms and 3 different maximum power outputs 50 W, 30 W and 15 Watts. For each load and maximum power output, measurements were repeated 3 times to assess the following parameters: (1) Maximum power output indicated on the DTA RFG; (2) Power output measured at the tip of the DTC; (3) Current measured at the tip of the DTC; (4) Peak-to-peak voltage measured at the tip of the DTC; (5) Maximum current variation among the 3 measurements.

### Accuracy assessment

The accuracy assessment of DTA visualization when using the Ensite X^TM^ EAM was assessed using a dedicated EAM phantom emulating the patient with patches connected. The phantom was similar to the one used during EAM installation prior to certification for clinical use. Different components were connected to the DTA RFG and EAM according to the different setup to be tested.

In NavX mode no geometry model was created; reference locations have been reached with ring electrode of Advisor HD Grid Mapping Catheter, Sensor Enabled (Abbott, St. Paul, MN) and tagged to create a baseline point set. Then DTA has been inserted into the phantom and its proper visualization has been visually assessed. Then the DTA has been moved to reference locations to ensure accuracy.

To assess the reliability of the catheter location on the EAM, the DTA was placed back at each reference location and an additional point was collected. An assessment was made regarding the reproducibility of the baseline point locations by measuring the distance between tags (in mm) on the EAM.

Each reinsertion and reconnection were performed 5 times prior to energy delivery. RF was delivered 3 times at each point.

For each setup, three different RF pulses were tested: (1) RF energy for 45 s with a target temperature of 60°C (longest application time and highest temperature allowed by the DTA in a clinical setting), with zero sec of power ramp delay, 1 s of pre-cooling and 0 s of cooling post ablation (RF1); or (2) RF energy for 10 s with a target temperature of 55°C (longest application time and highest temperature allowed by the DTA in a clinical setting) with zero sec of power ramp delay, 1 s of pre-cooling and 0 s of cooling post ablation (RF2); or (3) RF energy for 5 s with a target temperature of 50°C (longest application time and highest temperature allowed by the DTAS in a clinical setting) with zero sec of power ramp delay, 1 s of pre-cooling and 0 s of cooling post ablation (RF3).

Between each RF delivery the DTA was removed and reinserted in the phantom and the DTA cable was disconnected and re-connected without moving the catheter.

**Figure 5:**
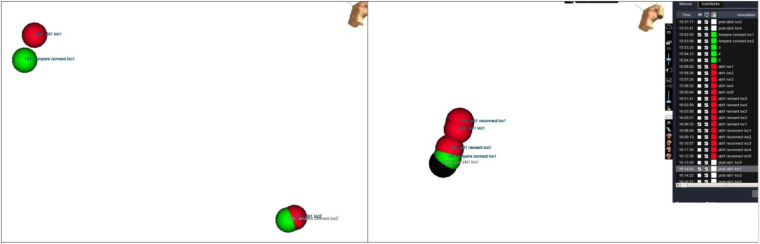
Accuracy measurements. Distance measurements between tags collected at same location during different moments of evaluation. Tip location has been tagged at each testing step and then distance has been measured.

After each RF application, the catheter was manually placed at each reference location by looking at the phantom directly. An additional point was collected at each reference location. Distance between every taken point and its corresponding baseline marker were measured using EAM software (Figure 5).

Distance measurements (in mm) between baseline points and collected points were repeated for every reference location on the phantom model after each RF pulse. At the next step, the DTA was placed once again back at each reference location and an additional final point was collected.

**Figure 6:**
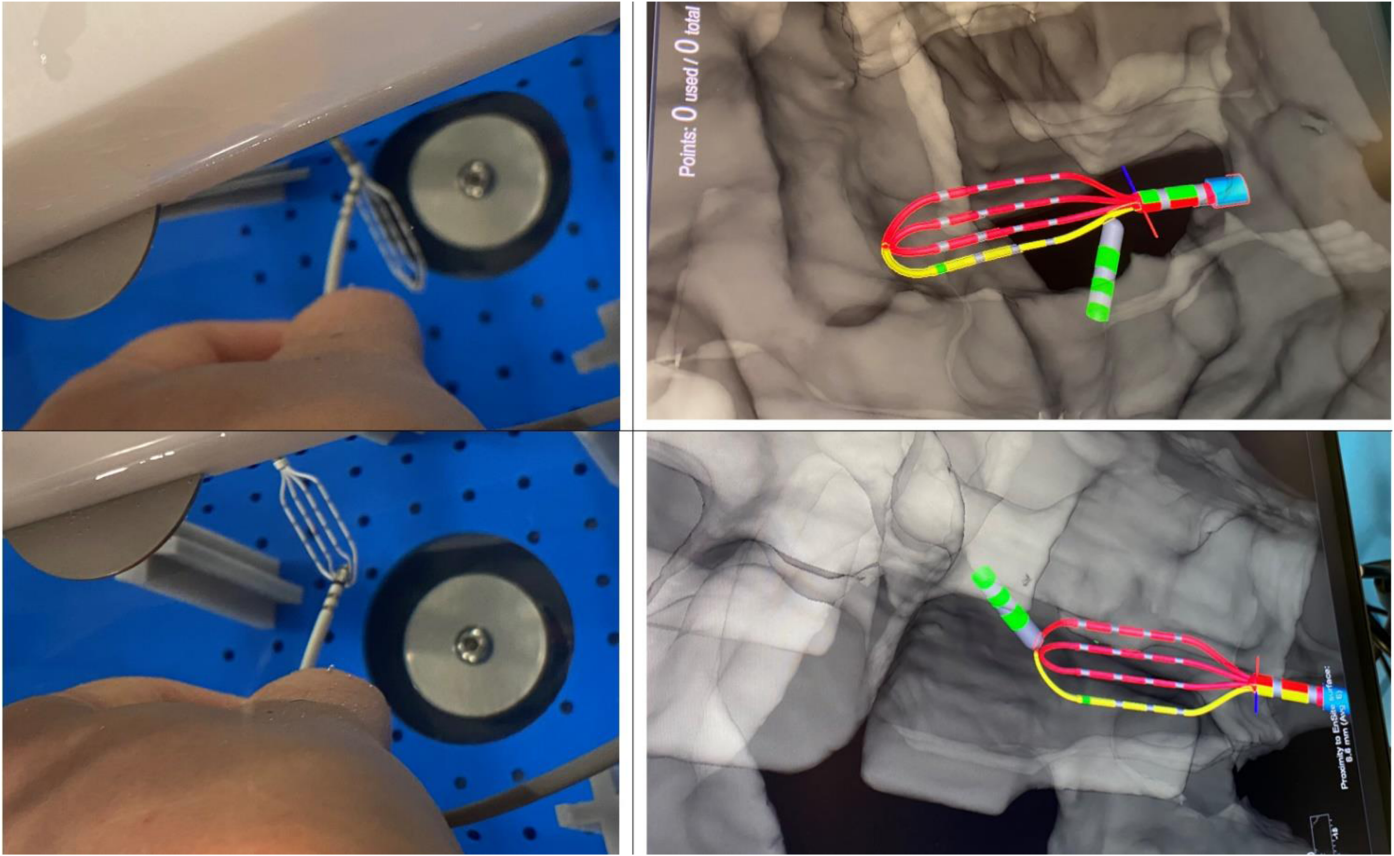
Visualization of DiamondTemp™ catheter in voXel mode. Photo of real location of HD-Grid mapping catheter and DiamondTemp™ (DTA) within the phantom and their representation on the Ensite System in Voxel mode.

Voxel mode testing has been performed only for configuration 3.2 as using configuration 3.1 in Voxel mode is not possible according to the instruction for use (IFU). The phantom anatomy was built using a dedicated mapping catheter, Advisor HD Grid Mapping Catheter, Sensor Enabled, (Abbott, St. Paul, MN). Reference locations from the phantom were reached with the tip of this catheter and reference locations were added to the surface of the phantom as baseline. Then the tracking of the DTA was verified by visual comparison of physical catheter movements and its representation on the map (Figure 6). The exact accuracy measurements in this mode were not possible as the system does not allow to tag locations from non-SE catheters.

### Configurations and settings of EnSiteX EAM and DTA

In configuration 3.1 DTA has been configured as an ablation catheter connected to the AmpereConnect. The original setup has been modified by connecting AmpereConnect cable between EAM and MDT GCB.

For configurations 3.2 and 3.3 the DTA has been configured as AUX catheter connected to the IC module of EnSite X and no modifications to the original setup has been done.

The accuracy assessment of the DTA visualization in the Voxel mode was based on the fact that Voxel mode uses a hybrid tracking mechanism. In this mode, the EAM system collects Voxels using SE catheter and aligns impedance values with magnetic points. This allows to accurately track non SE catheters.

### Statistical analysis

All variables were tested for normality with Shapiro–Wilk test. Normally distributed variables were described as mean ± standard deviation and the groups were compared through ANOVA, paired or unpaired t-test as appropriate, while the non-normally distributed variables were described as median (Inter Quartile Range) and compared by Kruskal-Wallis test, Mann-Whitney test or Wilcoxon signed-rank test as appropriate. The categorical variables were described as frequencies (percentages) and compared by Chi-squared test or Fisher's exact test as appropriate.

A *p*-value less than 0.05 was considered statistically significant.

The analysis was performed using R software version 3.6.2 (R Foundation for Statistical Computing, Vienna, Austria).

## Results

### DTA functional and safety parameters assessment with different setups

The data collected on the functional and safety parameters of the DTA connected to the EnSite X^TM^ EAM are detailed in Table 1 and Supplementary Table S1.

**Table 1:** Functional and safety parameters of DiamondTemp™ radiofrequency generator.

	Reference	Setup 3.1	Setup 3.2	Setup 3.3
15 Watt setting				
RFG Power Value (W)	15	15	15	15
Measured power (W)	15	15	15	15
Peak-to-Peak Voltage (V)	116	116	116	116
Current measured at tip (mA)	384	383	383	384
30 Watt setting				
RFG Power Value (W)	30	30	30	30
Measured power (W)	30	30	30	30
Peak-to-Peak Voltage (V)	164	164	164	165
Current measured at tip (mA)	542	541	541	542
50 Watt setting				
RFG Power Value (W)	50	50	50	50
Measured power (W)	50	50	50	50
Peak-to-Peak Voltage (V)	213	212	212	213
Current measured at tip (mA)	700	698	698	697

Measurements have been performed with 3 different loads (50 Ohm, 100 Ohm and 150 Ohm). Each measurement has been repeated 3 times. Data for 100 Ohm is shown in Table 1 (other measurements are available in Supplementary Table S1). *P* value = not significant (NS) for all comparisons.

At the lowest load setting of 50 Ohm, a maximum discrepancy of 5 W, could be observed between the maximum power programmed to be delivered by the DTA RFG, the actual power output indicated on the DTA RFG and the power output measured at the tip of the DTA.

Variations on the current measured at the tip of the DTA by RF analyzer could be observed between the 3 measurements performed with the same settings; All variations were within limits specified by the manufacturer of the device (7). The results are summarized in Table 1 and Supplementary Table S1.

### Accuracy of DTA visualization

The DTA location was represented in real-time for all configurations. A proper tracking of the DTA was observed by visual comparison of physical catheter movements and its representation on EAM. This was consistent with all setups tested.

Baseline points were taken using HD Grid Mapping Catheter tracked either using magnetic (Voxel mode) or impedance based (NavX mode) localization. The verification of the location of baseline points was established by taking points at the reference markers on the wet tank.

In setups 3.1 and 3.2 no major shifts were observed in the DTC location after RF1, RF2 or RF3 were performed with all setups tested, Table 2 and Supplementary Table S2. The location of the DTC did not significantly shift in space when compared to baseline reference points. This was consistent: after reinsertion and reconnection as well as following RF energy delivery. The distances measured between the baseline points after each variable, for each setup tested, are described in Table 2 and Supplementary Table S2.

**Table 2:** Accuracy of DiamondTemp^™^ visualization in EnsiteX system with different setups and different radiofrequency applications.

	Reference	Setup 3.1	Setup 3.2	Setup 3.3	*P* value
Distance after reinsertion (mm)	1.14 ± 0.4	1.49 ± 0.5	1.19 ± 0.1	5.16 ± 2.2	0.49/0.75/0.011
Distance after reconnection (mm)	1.26 ± 0.3	1.65 ± 0.7	1.44 ± 0.4	4.26 ± 1.6	0.37/0.38/0.013
Shift during RF (mm)	1.31 ± 0.2	1.35 ± 0.7	1.21 ± 0.3	13.81 ± 4.7	0.74/0.69/0.01
Shift after RF (mm)	1.09 ± 0.1	1.67 ± 0.6	1.12 ± 0.2	11.15 ± 3.2	0.31/0.68/0.002
Maximum shift observed (mm)	2.50 ± 0.4	4.00 ± 0.9	2.70 ± 0.6	21.40 ± 4.6	0.22/0.55/0.008

Each reinsertion and reconnection were performed 5 times prior to energy delivery. Radiofrequency (RF) was delivered 3 times at each point. The table summarizes average and maximal distance from catheter tip to the reference point after each step for 5 different points on the model. *P* value is reported for each comparison as follows: Reference-Setup 3.1/Reference-Setup 3.2/Reference-Setup 3.3. Details are available in Supplementary Table S2.

### Specific observations related to the setup 3.3

In this configuration a significant shift was observed immediately after catheter insertion. Catheter position was away from baseline points and this dislocation increased during the RF delivery. This may be linked to the fact that in this configuration no proper RF filtering is used. Due to huge baseline shift, further RF applications were not delivered as could be harmful for the EAM system with no added value as this configuration was suboptimal, Table 2 and Supplementary Table S2.

### Observations related to the setup 3.1 and 3.2

In these configurations no significant shift has been observed. All points were taken within the EnSiteX accuracy specifications (1), Table 2 and Supplementary Table S2.

### Specific observations related to voxel mode evaluation

Voxel mode evaluation has been performed only for the configurations 3.2 and 3.3 as per EnSite X IFU. Setup 3.3 showed a baseline shift in catheter position as in NavX mode. No RF application has been delivered in configuration 3.3 to avoid potential damage of the system.

Due to impossibility of adequately measuring accuracy in Voxel mode, a visual assessment of accuracy in Voxel mode has been performed. DTA has been placed on the surface of HDGrid and their relative visualizations on the EAM were compared. DTA was deemed as stable and accurate, Figure 6.

## Discussion

The main results of the current study are: (1) Previously developed universal method for compatibility assessment of ablation catheters and navigation systems has been used for a new EAM with reproducible results; (2) DTA is compatible with EnSiteX EAM. Safety and reliability profile is guaranteed within described specifications; (3) Careful setup is mandatory to achieve good clinical outcomes as only setup 3.2 is viable for both NavX and Voxel Mode and demonstrated satisfactory results.

3D mapping systems are considered as a standard of care for the diagnosis and treatment of cardiac arrythmias. They reduce radiation time and dose and improve the precision of ablation treatment (2, 5, 8). However, the use of third-party ablation catheters is limited as there is no compatibility out of the box. Extending the range of compatible ablation systems with various EAM allows new therapeutic modalities, which may be associated with clinical benefit.

The *in vitro* compatibility assessment is a crucial step, which must be done prior to clinical trials (3, 4). In the current study a previously developed universal method for compatibility assessment was used (1). It demonstrated to be reproducible with different EAM. This is of clinical relevance as it could be used for future standard bench evaluation before commercialization of novel components by different manufacturers.

Compared to our previous study, assessing compatibility between DTA and Rhythmia^TM^ EAM (Boston Scientific) (1), the current study is the first to evaluate the compatibility between DTA and another EAM, namely EnSiteX. The results showed that the same *in vitro* method can be applied to different EAM. Indeed, the experimental dataset hereby presented is completely new and this further reinforces the generalizability of the approach.

Furthermore, DTA has been demonstrated as compatible with EnSiteX EAM. Despite the current clinical use of DTA with EnSiteX EAM is feasible, configuration choice is of utmost importance. Indeed, safety and reliability of tracking is guaranteed within described settings. Both configurations 3.1 and 3.2 might be used for DTA in NavX mode. However only configuration 3.2 is possible for Voxel mode. If switching from NavX mode to Voxel mode can be required during procedure, careful pre procedural planning and proper connection setting should be considered.

## Limitations

This study is based on a phantom model. There was no test *in vivo*.

## Conclusions

Previously developed universal method for compatibility assessment of ablation catheters and navigation systems has been used for a new EAM with reproducible results. DTA is compatible with EnSiteX EAM with proper configuration.

## Data Availability

The original contributions presented in the study are included in the article/**Supplementary Material**, further inquiries can be directed to the corresponding author/s.
