# Supplementary material for: Compatibility assessment of a temperature-controlled radiofrequency catheter with a novel electroanatomical mapping system
Source: Front Cardiovasc Med. 2023 Apr 12;10:1086791. doi: 10.3389/fcvm.2023.1086791 (PMC10132728; doi:10.3389/fcvm.2023.1086791)
Supplement: Supplementary file 1 [file Table2.docx]

# SUpplementary materials

## Table S1. Functional and safety parameters of DiamondTemp^TM^ radiofrequency generator

### Functional and safety parameters observed with no external connections (reference) 1.0

| **Load (Ohm)** | **50** | | | **100** | | | **-** | | |
| --- | --- | --- | --- | --- | --- | --- | --- | --- | --- |
| **Max power programed on RFG (W)** | **50** | **30** | **15** | **50** | **30** | **15** | **50** | **30** | **15** |
| Power indicated on RFG during ablation (W) | 47 | 30 | 15 | 50 | 30 | 15 | 50 | 30 | 15 |
| Power measured at the tip of the DTC on the Electrosurgery analyzer (W) | 44 | 28 | 14 | 50 | 30 | 15 | 51 | 31 | 15 |
| Peak-to-peak voltage measured at the tip of the DTC on the Electrosurgery analyzer (V) | 138 | 110 | 77 | 213 | 164 | 116 | 262 | 203 | 143 |
| Current measured at the tip of the DTC on the Electrosurgery analyzer (mA) | 942 | 750 | 536 | 700 | 542 | 384 | 586 | 455 | 322 |
| Maximum variation in current measured at the tip of the DTC on the Electrosurgery analyzer between the 3 measurements performed (mA) | 0 | 0 | 0 | 0 | 0 | 0 | 0 | 0 | 0 |

### Functional and safety parameters observed with setup 3.1

| **Load (Ohm)** | **50** | | | **100** | | | **150** | | |
| --- | --- | --- | --- | --- | --- | --- | --- | --- | --- |
| **Max power programed on RFG (W)** | **50** | **30** | **15** | **50** | **30** | **15** | **50** | **30** | **15** |
| Power indicated on RFG during ablation (W) | 45 | 30 | 15 | 50 | 30 | 15 | 50 | 30 | 15 |
| Power measured at the tip of the DTC on the Electrosurgery analyzer (W) | 42 | 27 | 14 | 50 | 30 | 15 | 51 | 31 | 15 |
| Peak-to-peak voltage measured at the tip of the DTC on the Electrosurgery analyzer (V) | 135 | 109 | 77 | 212 | 164 | 116 | 261 | 202 | 143 |
| Current measured at the tip of the DTC on the Electrosurgery analyzer (mA) | 914 | 747 | 529 | 698 | 541 | 383 | 586 | 454 | 321 |
| Maximum variation in current measured at the tip of the DTC on the Electrosurgery analyzer between the 3 measurements performed (mA) | 0 | 0 | 0 | 0 | 0 | 0 | 0 | 0 | 0 |

### Functional and safety parameters observed with setup 3.2

| **Load (Ohm)** | **50** | | | **100** | | | **150** | | |
| --- | --- | --- | --- | --- | --- | --- | --- | --- | --- |
| **Max power programed on RFG (W)** | **50** | **30** | **15** | **50** | **30** | **15** | **50** | **30** | **15** |
| Power indicated on RFG during ablation (W) | 46 | 30 | 15 | 50 | 30 | 15 | 50 | 30 | 15 |
| Power measured at the tip of the DTC on the Electrosurgery analyzer (W) | 42 | 28 | 14 | 50 | 30 | 15 | 51 | 31 | 15 |
| Peak-to-peak voltage measured at the tip of the DTC on the Electrosurgery analyzer (V) | 135 | 109 | 77 | 212 | 164 | 116 | 261 | 203 | 143 |
| Current measured at the tip of the DTC on the Electrosurgery analyzer (mA) | 923 | 747 | 529 | 698 | 541 | 383 | 586 | 454 | 321 |
| Maximum variation in current measured at the tip of the DTC on the Electrosurgery analyzer between the 3 measurements performed (mA) | 4 | 0 | 0 | 0 | 0 | 0 | 0 | 0 | 0 |

### Functional and safety parameters observed with setup 3.3

| **Load (Ohm)** | **50** | | | **100** | | | **150** | | |
| --- | --- | --- | --- | --- | --- | --- | --- | --- | --- |
| **Max power programed on RFG (W)** | **50** | **30** | **15** | **50** | **30** | **15** | **50** | **30** | **15** |
| Power indicated on RFG during ablation (W) | 49 | 30 | 15 | 50 | 30 | 15 | 50 | 30 | 15 |
| Power measured at the tip of the DTC on the Electrosurgery analyzer (W) | 45 | 28 | 14 | 50 | 30 | 15 | 51 | 31 | 15 |
| Peak-to-peak voltage measured at the tip of the DTC on the Electrosurgery analyzer (V) | 141 | 110 | 77 | 213 | 165 | 116 | 262 | 203 | 143 |
| Current measured at the tip of the DTC on the Electrosurgery analyzer (mA) | 959 | 748 | 530 | 697 | 542 | 384 | 586 | 454 | 321 |
| Maximum variation in current measured at the tip of the DTC on the Electrosurgery analyzer between the 3 measurements performed (mA) | 10 | 0 | 0 | 0 | 0 | 0 | 0 | 0 | 0 |

## Table S2. Accuracy of DiamondTemp^TM^ visualization in EnsiteX system with different Setups and different radiofrequency applications

| ***Registered baseline points – with SE catheter – reference value*** | **Location 1** | **Location 2** | **Location 3** | **Location 4** | **Location 5** |
| --- | --- | --- | --- | --- | --- |
| *Distance to baseline points before any other testing variables (mm)* | NA | NA | NA | NA | NA |
| *Distance to baseline points after re-insertion of the ABL in the phantom (mm)* | 1.4 | 1.2 | 1.0 | 0.4 | 1.1 |
| *Distance to baseline points after re-connection of the ABL (mm)* | 1.3 | 1.7 | 2.5 | 1.1 | 1.1 |
|  |  | | |  |  |
| *Distance to baseline point at catheter location during RF1 delivery (mm)* | 1.5 | 1.0 | 1.2 | 1.4 | 1.5 |
| *Distance to baseline point after RF1 was performed (mm)* | 0.8 | 0.9 | 1.0 | 1.6 | 0.9 |
| *Distance to baseline point after reinserting the ABL following RF1 (mm)* | 1.3 | 1.7 | 1.5 | 0.5 | 0.9 |
| *Distance to baseline point at catheter location during 2nd RF1 delivery after re-inserting the ABL following the 1st RF1 delivery (mm)* | 1 | 1.5 | 1.5 | 1.5 | 1.2 |
| *Distance to baseline point after 2nd RF1 delivery, after re-inserting the DTC following the 1st RF1 delivery (mm)* | 0.8 | 1.6 | 1.0 | 1.7 | 1.2 |
| *Distance to baseline point after reconnecting the ABL following RF1 (mm)* | 1.2 | 1.2 | 1.5 | 0.9 | 1.0 |
| *Distance to baseline point at catheter location during 2nd RF1 delivery after reconnecting the DTC following the 1st RF1 delivery (mm)* | 1.0 | 1.1 | 0.9 | 1.5 | 1.4 |
|  |  | | |  |  |
| *Distance to baseline points after RF2 was performed (mm)* | No difference | No difference | No difference | No difference | No difference |
| *Distance to baseline points after reinserting the ABL following RF2 (mm)* | No difference | No difference | No difference | No difference | No difference |
| *Distance to baseline points after reconnecting the ABL following RF2 (mm)* | No difference | No difference | No difference | No difference | No difference |
|  |  | | |  |  |
| *Distance to baseline points after RF3 was performed (mm)* | No difference | No difference | No difference | No difference | No difference |
| *Distance to baseline points after reinserting the ABL following RF3 (mm)* | No difference | No difference | No difference | No difference | No difference |
| *Distance to baseline points after reconnecting the ABL following RF3 (mm)* | No difference | No difference | No difference | No difference | No difference |
|  |  | | |  |  |
| *Distance to baseline points after variables tested (mm)* | 1.8 | 1.5 | 1.2 | 1.5 | 1.1 |

| ***Registered baseline points for setup 3.1****.* | **Location 1** | **Location 2** | **Location 3** | **Location 4** | **Location 5** |
| --- | --- | --- | --- | --- | --- |
| *Distance to baseline points before any other testing variables (mm)* | No difference | No difference | No difference | No difference | No difference |
| *Distance to baseline points after re-insertion of the DTC in the phantom (mm)* | No difference | No difference | No difference | No difference | No difference |
| *Distance to baseline points after re-connection of the DTC (mm)* | No difference | No difference | No difference | No difference | No difference |
|  |  | | |  |  |
| *Distance to baseline point at catheter location during RF1 delivery (mm)* | 1.0 | 0.2 | 0.9 | 0.2 | 2.7 |
| *Distance to baseline points after reinserting the DTC following RF1 (mm)* | 2.0 | 1.3 | 1.0 | 1.2 | 2.3 |
| *Distance to baseline points after reconnecting the DTC following RF1 (mm)* | 1.7 | 1.5 | 0.9 | 1.3 | 1.8 |
| *Distance to baseline points at the end of the variables above following RF1 (mm)* | 1.4 | 1.8 | 1.1 | 1.1 | 1.5 |
|  |  | | |  |  |
| *Distance to baseline point at catheter location during RF2 delivery (mm)* | 1.1 | 1.7 | 1.0 | 1.3 | 3.9 |
| *Distance to baseline points after reinserting the DTC following RF2 (mm)* | 1.4 | 1.2 | 1.1 | 1.7 | 3.0 |
| *Distance to baseline points after reconnecting the DTC following RF2 (mm)* | 1.6 | 1.8 | 1.1 | 0.5 | 4.0 |
|  |  | | |  |  |
| *Distance to baseline points after RF3 was performed (mm)* | No difference | No difference | No difference | No difference | No difference |
| *Distance to baseline points after reinserting the DTC following RF3 (mm)* | No difference | No difference | No difference | No difference | No difference |
| *Distance to baseline points after reconnecting the DTC following RF3 (mm)* | No difference | No difference | No difference | No difference | No difference |
|  |  | | |  |  |
| *Distance to baseline points after variables tested (mm)* | 1.7 | 1.5 | 1.3 | 1.5 | 3.7 |

| ***Registered baseline points for setup 3.2*** | **Location 1** | **Location 2** | **Location 3** | **Location 4** | **Location 5** |
| --- | --- | --- | --- | --- | --- |
| *Distance to baseline points before any other testing variables (mm)* | No difference | No difference | No difference | No difference | No difference |
| *Distance to baseline points after re-insertion of the DTC in the phantom (mm)* | No difference | No difference | No difference | No difference | No difference |
| *Distance to baseline points after re-connection of the DTC (mm)* | No difference | No difference | No difference | No difference | No difference |
|  |  | | |  |  |
| *Distance to baseline point at catheter location during RF1 delivery (mm)* | 2.3 | 1.0 | 0.9 | 0.1 | 1.2 |
| *Distance to baseline points after RF1 was performed (mm)* | No difference | No difference | No difference | No difference | No difference |
| *Distance to baseline points after reinserting the DTC following RF1 (mm)* | 1.2 | 0.4 | 1.3 | 1.3 | 1.1 |
| *Distance to baseline points after reconnecting the DTC following RF1 (mm)* | 2.7 | 1.2 | 1.8 | 1.4 |  |
|  |  | | |  |  |
| *Distance to baseline points after RF2 was performed (mm)* | No difference | No difference | No difference | No difference | No difference |
| *Distance to baseline points after reinserting the DTC following RF2 (mm)* | No difference | No difference | No difference | No difference | No difference |
| *Distance to baseline points after reconnecting the DTC following RF2 (mm)* | No difference | No difference | No difference | No difference | No difference |
|  |  | | |  |  |
| *Distance to baseline points after RF3 was performed (mm)* | No difference | No difference | No difference | No difference | No difference |
| *Distance to baseline points after reinserting the DTC following RF3 (mm)* | No difference | No difference | No difference | No difference | No difference |
| *Distance to baseline points after reconnecting the DTC following RF3 (mm)* | No difference | No difference | No difference | No difference | No difference |
|  |  | | |  |  |
| *Distance to baseline points after variables tested (mm)* | 1.0 | 1.1 | 1.5 | 0.9 | 0.3 |

| ***Registered baseline points for setup 3.3*** | **Location 1** | **Location 2** | **Location 3** | **Location 4** | **Location 5** |
| --- | --- | --- | --- | --- | --- |
| *Distance to baseline points before any other testing variables (mm)* | NA | NA | NA | NA | NA |
| *Distance to baseline points after re-insertion of the DTC in the phantom (mm)* | 2.4 | 2.9 | 1.3 | 1.9 | 6.0 |
| *Distance to baseline points after re-connection of the DTC (mm)* | 2.3 | 1.7 | 2.5 | 4.0 | 5.8 |
|  |  | | |  |  |
| *Distance to baseline point at catheter location during RF1 delivery (mm)* | 13.5 | 14.0 | 11.2 | 8.4 | 20.5 |
| *Distance to baseline point after RF1 was performed (mm)* | 1.8 | 4.9 | 8.0 | 8.6 | 8.1 |
| *Distance to baseline point after reinserting the DTC following RF1 (mm)* | 2.3 | 4.7 | 6.5 | 8.5 | 8.1 |
| *Distance to baseline point at catheter location during 2nd RF1 delivery after re-inserting the DTC following the 1st RF1 delivery (mm)* | 15 | 13.5 | 12.5 | 8.5 | 21.2 |
| *Distance to baseline point after 2nd RF1 delivery, after re-inserting the DTC following the 1st RF1 delivery (mm)* | 1.8 | 4.6 | 6.0 | 8.7 | 8.6 |
| *Distance to baseline point after reconnecting the DTC following RF1 (mm)* | 1.2 | 5.2 | 9.5 | 8.9 | 8.0 |
| *Distance to baseline point at catheter location during 2nd RF1 delivery after reconnecting the DTC following the 1st RF1 delivery (mm)* | 16.0 | 13.1 | 9.9 | 8.5 | 21.4 |
|  |  | | |  |  |
| *Distance to baseline points after RF2 was performed (mm)* | No difference | No difference | No difference | No difference | No difference |
| *Distance to baseline points after reinserting the DTC following RF2 (mm)* | No difference | No difference | No difference | No difference | No difference |
| *Distance to baseline points after reconnecting the DTC following RF2 (mm)* | No difference | No difference | No difference | No difference | No difference |
|  |  | | |  |  |
| *Distance to baseline points after RF3 was performed (mm)* | No difference | No difference | No difference | No difference | No difference |
| *Distance to baseline points after reinserting the DTC following RF3 (mm)* | No difference | No difference | No difference | No difference | No difference |
| *Distance to baseline points after reconnecting the DTC following RF3 (mm)* | No difference | No difference | No difference | No difference | No difference |
|  |  | | |  |  |
| *Distance to baseline points after variables tested (mm)* | 1.8 | 3.5 | 5.2 | 8.5 | 3.5 |
